# Antidepressant drug treatment protecting from COVID-19: one more piece in the repurposing puzzle

**DOI:** 10.1192/bjo.2021.1075

**Published:** 2021-12-20

**Authors:** Julia C. Stingl

**Affiliations:** Institute of Clinical Pharmacology, University Hospital of RWTH Aachen, Germany.

**Keywords:** Antidepressants, repurposing, SSRI, COVID-19, prevention

## Abstract

In the article Analysis of the impact of antidepressants and other medications on COVID-19 infection risk in a chronic psychiatric in-patient cohort, Catherine L. Clelland and colleagues for the first time suggest a protective effect of antidepressants against infection with coronavirus disease 2019 (COVID-19) itself. During the observation period of the first wave of the pandemic in New York, more than 50% of patients in the psychiatric hospital studied were infected. From retrospective analysis of the hospital medical records, the authors found a significantly lower risk for infection in patients with antidepressant medication compared to treatment with other psychiatric drugs. The findings of a reduced infection incidence in patients who were already on antidepressant drug therapy underlines a preventive efficacy of antidepressants against COVID-19. Taken together with the prior obtained data of efficacy against deterioration of COVID-19 disease, this study adds a piece of evidence to the positive benefit-risk of antidepressants in repurposing condition against COVID-19.

## Background

In the article Analysis of the impact of antidepressants and other medications on COVID-19 infection risk in a chronic psychiatric in-patient cohort, Catherine L. Clelland and colleagues report on the potential protective effect of antidepressants against infection with coronavirus disease 2019 (COVID-19).^1^ Their retrospective cohort study was conducted in an in-patient setting at a large hospital in New York where it appears reasonable to assume that virus exposure had been relatively uniform across the facility and antidepressant drug intake in patients was common. It adds to the increasing body of knowledge about the potential efficacy of antidepressants against severe courses of SARS-CoV-2 infections, with the finding of a reduced infection incidence in patients who were already on antidepressant drug therapy.

## Impact on disease course

Former observational studies had reported a more favourable COVID-19 disease course, such as a reduced risk of intubation or death, in patients treated with serotonin reuptake inhibitors or serotonin-2 antagonist and reuptake inhibitors.^2–4^ A small randomised placebo-controlled trial testing 15 days of fluvoxamine in COVID-19-infected patients found a significant effect on disease deterioration, with no cases of deterioration (0/80) in the fluvoxamine arm compared with 8.3% (6/72) in the placebo arm.^5^ Another prospective cohort study with fluvoxamine also showed a preventive effect against deterioration in the patients treated with fluvoxamine.^6^ In addition, a large randomised multicentre placebo-controlled TOGETHER trial showed significant benefit of fluvoxamine in *n* = 1472 patients with COVID-19 infection, preventing severe complications.^7^ Another clinical open-label trial with fluvoxamine in *n* = 51 intensive care unit-treated patients showed a 42% reduction in mortality compared with matched controls.^8^

## A protective effect against infection

The present study for the first time suggests a protective effect even against the infection itself. During the observation period of the first wave of the pandemic in New York, more than 50% of the patients in the psychiatric hospital of the study were infected. By retrospectively analysing medical records, the authors found significantly lower odds ratios for infection risk in patients taking antidepressant medication compared with other psychiatric drugs.

The potential protective effect was shown over all antidepressant classes, but subgroup analyses showed that especially patients treated with serotonin reuptake inhibitors and serotonin–norepinephrine reuptake inhibitors or serotonin-2 antagonist reuptake inhibitors had significantly decreased COVID-19 infections. The association with specific substances of these antidepressant drug classes may be as a result of the relative frequency with which they are prescribed. Indeed, preclinical studies have shown that uptake of SARS-CoV-2 is blocked by all antidepressant drug classes (also tricyclic and tetracyclic).^9^

## Potential explanation for these findings

As this was a retrospective analysis, the outcomes of infection have not been followed up. However, the findings of not only less severe courses of COVID-19 disease but also of a lower risk for infection support the hypothesis of potential antiviral protective activity provided by the antidepressant drugs analysed in the study. This hypothesis has been advanced by *in vitro* analyses showing that antidepressants may possess antiviral properties through different mechanisms of action.

First, antidepressants have been shown to interact with the sphingomyelinase/ceramide system, which is likely required to facilitate angiotensin-converting enzyme 2 (ACE2) binding of the SARS-CoV-2 and thereby impair viral host cell entry.^10–12^ In addition to antidepressant drugs, several medications share the property of functional inhibition of the acid sphingomyelinase. Recent observational data show a reduced likelihood of intubation or death in (*n* = 2846) COVID-19-infected patients who are treated with any of the functional sphingomyelinase/ceramide system inhibitory drugs.^13^

Second, antidepressants may exert anti-inflammatory activity, by reducing levels of proinflammatory cytokines including interleukin (IL-6), IL-10, tumour necrosis factor (TNF)-alpha, and chemokine (C-C motif) ligand (CCL)-2 or by activating the sigma-1 receptor.^14–17^

Third, serotonin uptake inhibitors may reduce platelet activity by decreasing serotonin levels in platelets. This may positively affect the course of COVID-19 disease and counteract the serotonin syndrome that seems to occur in some of these patients.^18^

In addition to the pharmacological properties of the antidepressant drugs, people with depression tend to avoid social contacts. During the pandemic, depression-induced social isolation may have prevented viral exposure and COVID illness. However, there was no difference in COVID-19 infection status between patients with manic and depressive symptoms in the study by Clelland. Hence, confounding by indication for the medication appears unlikely.

## Conclusions

In the presence of already substantial evidence for an association of antidepressant drug use with better outcomes in patients with a severe and laboratory-confirmed COVID-19 infection, this study highlights the potentially underestimated finding of a protective effect of antidepressant drug use (such as the selective serotonin reuptake inhibitor fluoxetine or the serotonin-2 antagonist and reuptake inhibitor trazodone) against the infection itself in patients who are being treated with antidepressants for depression and/or other psychiatric disorders. This is especially worth mentioning because antidepressant drug use is common in a substantial proportion of the general population. It is possible that the inclusion of severe cases in epidemiological studies (i.e. only patients with laboratory-confirmed COVID-19 infection) may contribute to this underestimation. Taken together with the prior evidence, this study underlines the benefit of using antidepressant drugs to prevent COVID-19 infection and deterioration of COVID-19 disease.

Taken together with the prior evidence, this study underlines the benefit of using certain antidepressant drugs, such as the selective serotonin reuptake inhibitor fluoxetine or the serotonin-2 antagonist and reuptake inhibitor trazodone, to prevent COVID -19 infection and deterioration of COVID-19 disease.

## References

[1] Clelland C, Ramiah K, Steinberg L, Clelland J. Analysis of the impact of antidepressants and other medications on COVID-19 infection risk in a chronic psychiatric in-patient cohort. BJPsych Open 2021; 8: E6. 34859759 10.1192/bjo.2021.1053 PMC8649363

[2] Hoertel N, Sanchez-Rico M, Vernet R, Beeker N, Jannot AS, Neuraz A, Association between antidepressant use and reduced risk of intubation or death in hospitalized patients with COVID-19: results from an observational study. Mol Psychiatry 2021; 26: 5199–212. 33536545 10.1038/s41380-021-01021-4

[3] Diez-Quevedo C, Iglesias-Gonzalez M, Giralt-Lopez M, Rangil T, Sanagustin D, Moreira M, Mental disorders, psychopharmacological treatments, and mortality in 2150 COVID-19 Spanish inpatients. Acta Psychiatr Scand 2021; 143 526–34. 33792912 10.1111/acps.13304 PMC8250711

[4] Oskotsky T, Maric I, Tang A, Oskotsky B, Wong RJ, Aghaeepour N, Mortality risk among patients with COVID-19 prescribed selective serotonin reuptake inhibitor antidepressants. JAMA Netw Open 2021; 4: e2133090. 34779847 10.1001/jamanetworkopen.2021.33090 PMC8593759

[5] Lenze EJ, Mattar C, Zorumski CF, Stevens A, Schweiger J, Nicol GE, Fluvoxamine vs placebo and clinical deterioration in outpatients with symptomatic COVID-19: a randomized clinical trial. JAMA 2020; 324: 2292–300. 33180097 10.1001/jama.2020.22760 PMC7662481

[6] Seftel D, Boulware DR. Prospective cohort of fluvoxamine for early treatment of coronavirus disease 19. Open Forum Infect Dis 2021; 8: ofab050. 33623808 10.1093/ofid/ofab050 PMC7888564

[7] Reis G, Dos Santos Moreira-Silva EA, Silva DCM, Thabane L, Milagres AC, Ferreira TS, Effect of early treatment with fluvoxamine on risk of emergency care and hospitalisation among patients with COVID-19: the TOGETHER randomised, platform clinical trial. Lancet Glob Health [Epub ahead of print] 27 Oct 2021. Available from: 10.1016/S2214-109X(21)00448-4. PMC8550952 34717820

[8] Calusic M, Marcec R, Luksa L, Jurkovic I, Kovac N, Mihaljevic S, Safety and efficacy of fluvoxamine in COVID-19 ICU patients: an open label, prospective cohort trial with matched controls. Br J Clin Pharmacol [Epub ahead of print] 1 Nov 2021. Available from: 10.1111/bcp.15126. PMC8653355 34719789

[9] Carpinteiro A, Edwards MJ, Hoffmann M, Kochs G, Gripp B, Weigang S, Pharmacological inhibition of acid sphingomyelinase prevents uptake of SARS-CoV-2 by epithelial cells. Cell Rep Med 2020; 1: 100142. 33163980 10.1016/j.xcrm.2020.100142 PMC7598530

[10] Hoertel N, Sanchez-Rico M, Cougoule C, Gulbins E, Kornhuber J, Carpinteiro A, Repurposing antidepressants inhibiting the sphingomyelinase acid/ceramide system against COVID-19: current evidence and potential mechanisms. Mol Psychiatry [Epub ahead of print] 2 Aug 2021. Available from: 10.1038/s41380-021-01254-3. PMC8359627 34385600

[11] Kornhuber J, Hoertel N, Gulbins E. The acid sphingomyelinase/ceramide system in COVID-19. Mol Psychiatry [Epub ahead of print] 4 Oct 2021. Available from: 10.1038/s41380-021-01309-5. PMC8359627 34385600

[12] Marin-Corral J, Rodriguez-Morato J, Gomez-Gomez A, Pascual-Guardia S, Munoz-Bermudez R, Salazar-Degracia A, Metabolic signatures associated with severity in hospitalized COVID-19 patients. Int J Mol Sci 2021; 22; 4794. 10.3390/ijms22094794 PMC8124482 33946479

[13] Hoertel N, Sanchez-Rico M, Gulbins E, Kornhuber J, Carpinteiro A, Lenze EJ, Association between FIASMAs and reduced risk of intubation or death in individuals hospitalized for severe COVID-19: an observational multicenter study. Clin Pharmacol Ther 2021; 110: 1498–511. 34050932 10.1002/cpt.2317 PMC8239599

[14] Ishima T, Fujita Y, Hashimoto K. Interaction of new antidepressants with sigma-1 receptor chaperones and their potentiation of neurite outgrowth in PC12 cells. Eur J Pharmacol 2014; 727: 167–73. 24508523 10.1016/j.ejphar.2014.01.064

[15] Rosen DA, Seki SM, Fernandez-Castaneda A, Beiter RM, Eccles JD, Woodfolk JA, Modulation of the sigma-1 receptor-IRE1 pathway is beneficial in preclinical models of inflammation and sepsis. Sci Transl Med 2019; 11; eaau5266. 10.1126/scitranslmed.aau5266 PMC6936250 30728287

[16] Kohler CA, Freitas TH, Stubbs B, Maes M, Solmi M, Veronese N, Peripheral alterations in cytokine and chemokine levels after antidepressant drug treatment for major depressive disorder: systematic review and meta-analysis. Mol Neurobiol 2018; 55: 4195–206. 28612257 10.1007/s12035-017-0632-1

[17] Roumestan C, Michel A, Bichon F, Portet K, Detoc M, Henriquet C, Anti-inflammatory properties of desipramine and fluoxetine. Respir Res 2007; 8: 35. 17477857 10.1186/1465-9921-8-35 PMC1876225

[18] Sukhatme VP, Reiersen AM, Vayttaden SJ, Sukhatme VV. Fluvoxamine: a review of its mechanism of action and its role in COVID-19. Front Pharmacol 2021; 12: 652688. 33959018 10.3389/fphar.2021.652688 PMC8094534

